# Hemodynamic Imaging in Cerebral Diffuse Glioma—Part B: Molecular Correlates, Treatment Effect Monitoring, Prognosis, and Future Directions

**DOI:** 10.3390/cancers14051342

**Published:** 2022-03-05

**Authors:** Vittorio Stumpo, Lelio Guida, Jacopo Bellomo, Christiaan Hendrik Bas Van Niftrik, Martina Sebök, Moncef Berhouma, Andrea Bink, Michael Weller, Zsolt Kulcsar, Luca Regli, Jorn Fierstra

**Affiliations:** 1 Department of Neurosurgery, University Hospital Zurich, 8091 Zurich, Switzerland; leliog06@gmail.com (L.G.); jacopo.bellomo@usz.ch (J.B.); bas.vanniftrik@usz.ch (C.H.B.V.N.); martina.seboek@usz.ch (M.S.); luca.regli@usz.ch (L.R.); jorn.fierstra@usz.ch (J.F.); 2 Clinical Neuroscience Center, University Hospital Zurich, University of Zurich, 8057 Zurich, Switzerland; andrea.bink@usz.ch (A.B.); michael.weller@usz.ch (M.W.); zsolt.kulcsar@usz.ch (Z.K.); 3 Department of Neurosurgical Oncology and Vascular Neurosurgery, Pierre Wertheimer Neurological and Neurosurgical Hospital, Hospices Civils de Lyon, 69500 Lyon, France; moncef.berhouma@chu-lyon.fr; 4 Department of Neuroradiology, University Hospital Zurich, 8091 Zurich, Switzerland; 5 Department of Neurology, University Hospital Zurich, 8091 Zurich, Switzerland

**Keywords:** hemodynamic, diffuse glioma, glioblastoma, molecular features, perfusion MRI, cerebrovascular reactivity, tumor progression, radiation necrosis, prognosis, radiomics, machine learning

## Abstract

**Simple Summary:**

Cerebral diffuse gliomas present peculiar molecular features tightly linked to phenotypic characteristics that are not readily appreciated by means of standard neuroimaging. In the present Part B of our two-review series, the potential of exploiting glioma vascular and hemodynamic alterations for a better characterization of tumor subtype, differentiation of tumor recurrence from treatment effects, and prognosis prediction is critically discussed together with the advancements related to radiomics and machine learning for innovative imaging biomarkers development.

**Abstract:**

Gliomas, and glioblastoma in particular, exhibit an extensive intra- and inter-tumoral molecular heterogeneity which represents complex biological features correlating to the efficacy of treatment response and survival. From a neuroimaging point of view, these specific molecular and histopathological features may be used to yield imaging biomarkers as surrogates for distinct tumor genotypes and phenotypes. The development of comprehensive glioma imaging markers has potential for improved glioma characterization that would assist in the clinical work-up of preoperative treatment planning and treatment effect monitoring. In particular, the differentiation of tumor recurrence or true progression from pseudoprogression, pseudoresponse, and radiation-induced necrosis can still not reliably be made through standard neuroimaging only. Given the abundant vascular and hemodynamic alterations present in diffuse glioma, advanced hemodynamic imaging approaches constitute an attractive area of clinical imaging development. In this context, the inclusion of objective measurable glioma imaging features may have the potential to enhance the individualized care of diffuse glioma patients, better informing of standard-of-care treatment efficacy and of novel therapies, such as the immunotherapies that are currently increasingly investigated. In Part B of this two-review series, we assess the available evidence pertaining to hemodynamic imaging for molecular feature prediction, in particular focusing on isocitrate dehydrogenase (IDH) mutation status, MGMT promoter methylation, 1p19q codeletion, and EGFR alterations. The results for the differentiation of tumor progression/recurrence from treatment effects have also been the focus of active research and are presented together with the prognostic correlations identified by advanced hemodynamic imaging studies. Finally, the state-of-the-art concepts and advancements of hemodynamic imaging modalities are reviewed together with the advantages derived from the implementation of radiomics and machine learning analyses pipelines.

## 1. Introduction

**Figure 1:**
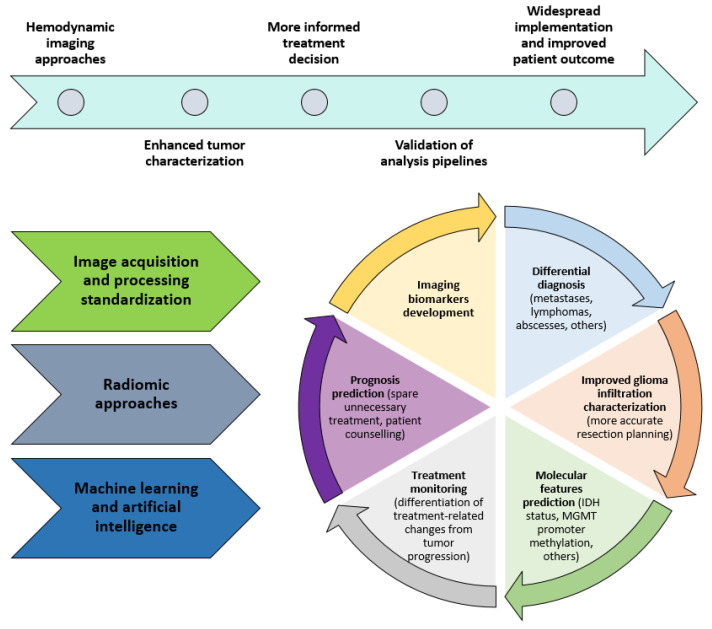
Schematic representing the overview of hemodynamic imaging past progress and future potential for enhanced glioma characterization.

In recent decades, the traditional histopathological grading of diffuse gliomas has been complemented by a more refined molecular analysis of tumor markers. Gliomas, and glioblastomas in particular, exhibit an extensive intra- and inter-tumoral molecular heterogeneity which represent complex biological features, correlating to the efficacy of treatment response and survival [1]. This broadened understanding has translated into a more comprehensive WHO classification of high grade gliomas [2] as well as more tailored treatment guidelines [3]. Importantly, from a neuroimaging point of view, these specific molecular and histopathological features may be used to yield imaging biomarkers as surrogates for distinct tumor genotypes and phenotypes [4]. The development of comprehensive glioma imaging markers has potential for improved glioma characterization that will assist in the clinical work-up of preoperative treatment planning and treatment effect monitoring. Current imaging techniques are yet to overcome certain limitations which, until now, have prohibited a clear demarcation of the diffuse glioma infiltration zone as well as the molecular phenotypes present within the lesion. These aspects are of particular clinical relevance since the differentiation of tumor recurrence or true progression from pseudoprogression, pseudoresponse, and radiation-induced necrosis are still not reliably determined through standard neuroimaging only [5]. Given the abundant vascular and hemodynamic alterations present in diffuse glioma, advanced hemodynamic imaging approaches constitute an attractive area of clinical imaging development (Figure 1).

Hemodynamic imaging of brain tumors adopts several techniques, including perfusion MRI sequences, such as dynamic susceptibility contrast (DSC)-MRI, dynamic contrast-enhanced (DCE)-MRI, arterial spin labelling (ASL)-MRI and perfusion computed tomography (PCT) together with a number of other advanced hemodynamic imaging approaches that are increasingly investigated. A description of hemodynamic imaging modalities and relative assessed parameters is presented in Part A of the present review (see Tables 1 and 2 of Part A) [6].

In this context, the inclusion of objective measurable glioma imaging features may have the potential to enhance the individualized care of diffuse glioma patients, which can better inform standard-of-care treatment efficacy and novel therapies, such as immunotherapies that are currently being increasingly investigated [7]. In Part B of this review, we assess the available evidence pertaining to hemodynamic imaging for molecular feature prediction. The results on the differentiation of tumor progression/recurrence due to treatment effects have also been the focus of active research and are presented together with the prognostic correlations identified by advanced hemodynamic imaging studies. Finally, state-of-the-art concepts and advancements in hemodynamic imaging modalities are reviewed together with the advantages derived by the implementation of radiomics and the machine learning analyses pipelines.

## 2. Clinical Applications of Hemodynamic Imaging in Gliomas—Part 2

### 2.1. Molecular Features Prediction

With the increasingly understood role of biology in correlating tumor aggressiveness and prognosis, the old histological entities are now outdated and tumor classification depends on underlying molecular features [2]. This information, which is crucial in patient management, is currently only available after the analysis of tumor specimens (biopsy/resection). In this respect, advancements in neuroimaging can play a determinant role in inferring the tumor characteristics, metabolism, and micro-structure [8] but they also provide additional means of correlating the imaging biomarkers to the tumor molecular features pre-operatively, i.e., radiogenomics [9]. Examples of efforts in this direction include the availability of the Cancer Genome Atlas imaging datasets through the Cancer Imaging Archive (https://www.cancerimagingarchive.net/ Last accessed on 21 December 2021). More importantly, this hypothesis has been also extensively explored in hemodynamic imaging [10,11,12,13,14,15,16,17,18,19,20,21,22,23,24,25,26,27,28,29,30,31,32,33,34,35]. In this section, we focus in particular on hemodynamic correlates of isocitrate dehydrogenase (IDH) mutation status, 1p19q codeletion, O^6^-methylguanine-DNA methyl-transferase (MGMT) promoter methylation, and epidermal growth factor receptor (EGFR) alterations.

### 2.2. IDH Mutation Status

IDH mutations are the most important prognostic factors according to the present molecular classification of gliomas whereby the absence of mutation (mut), i.e., IDH wildtype (wt) tumors, is characterized by a worse prognosis. IDH1/2 mutations occurring in the catalytic pocket generate a neomorphic enzyme that converts α-ketoglutarate and NADPH to (R)-2hydroxyglutarate (2-HG). The IDHmut and the accumulation of the “oncometabolite” 2-HG are associated with the hypermethylation phenotype, called glioma CpG island methylator, ultimately altering metabolism to promote malignant growth [36,37]. As the prognostic implications of reliable pre-operative identification of this molecular marker could significantly alter the clinical management decisions including the radicality of the surgical resection, several studies have investigated the potential for IDH status prediction in diffuse gliomas using hemodynamic imaging [10,11,20,29,30,32,38]. Consistently with “traditional” tumor grading, IDHmut gliomas tend to exhibit decreased perfusion and permeability with respect of their wild-type counterpart. Lower DSC-cerebral blood volume(CBV) [10,20,29,32,39,40], as with lower DSC-cerebral blood flow (CBF) [30,38], were found to be able to discriminate IDHmut versus IDHwt. Percentage of signal recovery (PSR) was also reported to be lower in IDHwt tumors [40]. DCE studies assessing the permeability parameters also showed potential for distinguishing between the two molecular entities. IDHmut gliomas were found to exhibit decreased CBF, volume transfer constant between blood plasma and extravascular extracellular space (Ktrans), blood plasma fractional volume (Vp), extravascular extracellular volume fraction (Ve), and area under the curve (AUC) [11,41,42]. With respect of ASL-CBF, some authors like Brendle et al. and Yoo et al. reported it to be useful to distinguish between these two subgroups [43,44], while others found only a moderate correlation [12]. Consistently with these findings, PCT studies have also described decreased CBV and permeability surface area product (PS) in IDHmut gliomas [45]. These hemodynamic findings altogether support the concept of IDHmut tumoral tissue with reduced microvascular density and permeability with respect of IDHwt. This is consistent with their decreased aggressiveness and reduced activation of the hypoxic-angiogenetic pathway [29,46].

### 2.3. p/19q Codeletion

Oligodendroglioma is genetically defined as a tumor harboring a IDH1/IDH2 mutation involving the co-deletion of chromosome arms 1p and 19q. Diagnosis can be obtained only using pathological tissue after a biopsy or resection [3,47] but hemodynamic imaging has also shown potential for the identification of IDH 1p/19q codeletion status as part of the differentiation of oligodendroglioma from the IDH mutant 1p/19q non-deleted astrocytomas and IDHwt glioblastomas [10,11,12,30,48]. The pre-operative identification of 1p/19q is clinically relevant as oligodendrogliomas are characterized by a lower impact of the extent of resection with respect of IDHmut astrocytoma, whereas they also display a better response to cytotoxic agents and increased OS [49]. DSC-CBV has been found to be increased in IDHmut 1p/19q codel grade 3 gliomas as opposed to IDHmut 1p/19q nodel grade 3, [30,48,50,51] suggesting denser and more heterogenous vascular distribution in the former [48], but decreased with respect to IDHwt glioblastomas [10,50,52]. In a meta-analysis by Delgado and Delgado investigating whether DSC can differentiate between grade 2 and 3 gliomas, diagnostic accuracy was substantially decreased for differentiating between oligodendroglioma grades 2 and 3 [53]. With respect of DCE, Conte et al. found that there were no difference in DCE parameters when distinguishing between IDHmut gliomas with or without 1p/19qdel [11] in line with the findings of Yoon et al. [52]. These instead reported Ktrans and Ve to be significantly decreased in grade 2–3 oligodendrogliomas compared with glioblastomas [52]. On the contrary, a study by Lee et al. using histogram analysis reported increased Ktrans, Ve in grade 2–3 oligodendrogliomas versus astrocytomas grade 2–3 [51]. ASL-derived CBF used to predict IDH genotype and 1p/19 codel, despite showing a moderate correlation in some studies, failed to reach a significant association in others [12,44]. PCT studies reported a higher CBV and lower PS in 1p/19qcodel gliomas compared with the intact counterparts [54].

### 2.4. MGMT Promoter Methylation

MGMT promoter methylation has drawn considerable attention as its presence contributes to the response to temozolomide. A selected population of MGMT unmethylated glioblastoma patients may reach a comparable outcome when chemotherapy is withhold [55]. Due to its potential to drive management decisions, a number of studies have investigated the possibility to pre-operatively identify MGMT promoter methylation through hemodynamic imaging [31,34,35,56,57] despite some investigations having reported histopathological and immunohistochemical analysis not differing significantly between these two phenotypes [58]. In line with the concept so far explored, indicating that increased tumor perfusion correlates to aggressiveness, MGMT methylated gliomas have also been found to exhibit decreased CBV [59,60,61] as well as decreased peak height (PH) [59]. CBF differences were not found in DSC studies [60]. Nevertheless, the evidence is conflicting as a previous smaller series by Moon et al. observed no significant difference in CBV [56]. The results were also confirmed in a larger study population described by Fuster-Garcia et al. [62]. Interestingly, a recent study by Choi et al. showed that DSC-CBV in a non-enhancing tumor could be used to predict methylation status change at recurrence [35]. Similar conflicting evidence has been reported in DCE studies. Zhang et al. utilized DCE-parameters in a histogram analysis and found increased Ve and Ktrans in MGMT unmethylated tumors [40]. These findings contradict a previous study by Ahn et al. who on the contrary reported reduced Ktrans in MGMT unmethylated tumors [31]. Further complicating the matter, one study reported that ASL-CBF in CE was found to be to be significantly higher in unmethylated versus methylated gliomas [43].

### 2.5. EGFR Mutation

Following the detection of epidermal growth factor receptor (EGFR) gene alterations (such as amplifications, mutations, and translocation) in a high percentage of glioblastomas, the attempt to develop treatment strategies targeting EGFR has been pursued [63]. Given the availability of receptor-tyrosine kinase inhibitors and the possibility of testing these drugs in clinical trials (with at present disappointing results [64]), the non-invasive imaging of EGFR alteration is clinically relevant and hemodynamic imaging approaches have been attempted [26,33,65,66,67]. DSC-CBV was reported to be significantly higher in EGFRvIII expressing tumors than in wild-type ones [67]. Gupta et al. reported a higher CBV (finding not confirmed by Oughourlian et al. [68]) and lower PSR in tumors with EGFR amplification, while EGFRvIII mutated tumors were associated with higher PH [65]. DCE studies found higher Vp and Ktrans, with the former histogram metrics outperforming the latter for this aim [66]. A study by Qiao et al. found there to be a significant association between a ASL-CBF hypervascular pattern identified with high inter-rater agreement and EGFRvIII expression [33]. 

### 2.6. Other Markers: Hypoxia, Angiogenesis, Proliferation

Given the relationship of aberrant glioblastoma vasculature with hypoxia playing a role in its development and invasion, a number of studies have investigated the potential for correlating the perfusion measures to hypoxic and angiogenic markers [16,18,21,23,24,25,26,27,28,69,70]. In parallel, markers of cellular proliferation—indicating tumor malignancy—such as Ki67 have been correlated to perfusion parameters [14,15,28]. The genetic heterogeneity characteristic of diffuse cerebral glioma results in deregulated molecular pathways whose more relevant effectors such as TERT, ATRX, PTEN, and mTOR have also been correlated to perfusion imaging [40,59,71]. 

### 2.7. Differentiation between Tumor Progression/Tumor Recurrence vs Radiation Necrosis/Pseudoprogression/Pseudoresponse

An additional clinically relevant limitation of the current standard imaging is a suboptimal differentiation between tumor progression/recurrence and radionecrosis, pseudoprogression, or pseudoresponse [72]. Pseudoprogression, with a reported variable incidence rate of 10 to 30% in patients receiving chemoradiotherapy, is defined by the presence of new or enlarging area(s) of contrast-enhancement in the absence of true tumor growth which regresses or stabilizes despite no changes in treatment [73]. Due to its similarities with the other above-mentioned entities, diagnosis is usually retrospective but it can be also obtained by the tissue sample analysis. Pathophysiologically, this phenomenon is likely to be determined by the transiently increased capillary permeability of pathological vasculature as well as by the inflammation determined through chemoradiotherapy. The difference to radiation necrosis is related both to the timing of presentation, i.e., 3–6 months for pseudoprogression and 1 year after radiation therapy in radiation necrosis, but also to the different pathophysiology whereby radiation necrosis presents as permanent damage to the brain tissue, necrosis, and vascular thrombosis [74]. Variable “timing” definitions and the blurred presentation between these two entities reported in published studies have important implications in terms of patient management, clinical trial enrolment, and treatment evaluation [75]. In fact, patients with pseudoprogression are notably characterized by a favorable clinical course. On the opposite spectrum, falsely favorable imaging signs are present in pseudoresponse whereby the use of antiangiogenic treatment, i.e., VEGF inhibitors induce the normalization of the BBB, thus leading to a reduction in contrast enhancement and edema in the T2/FLAIR sequence. In this case, the observed improvement is a sole consequence of the alterations in vascular permeability and is thus unrelated to treatment efficacy [5]. Some other chemotherapeutic treatments can also induce imaging abnormalities and an accurate distinction from relapse is therefore needed [76]. Several studies have addressed this clinical issue [76,77,78,79,80,81,82,83,84,85,86,87,88,89,90,91,92,93,94,95,96,97,98,99]. As for the other previously presented outcomes, DSC has been more extensively investigated. A number of research groups confirmed DSC-CBV to be higher in tumor recurrence/progression versus radiation necrosis [78,79,81,82,83,89,91,93,94,97,100,101,102,103]. These results have been accordingly strengthened by meta-analyses [104,105,106,107]. Chuang et al. in 2016 performed a random effect model meta-analysis of ten studies evaluating CBV and found that this parameter is increased in the contrast-enhancing lesion of tumor recurrence versus radiation injury [105]. Another meta-analysis by Patel et al. found that after selecting the best performing parameter from each study, the pooled sensitivity and specificity were 0.90 and 0.88 respectively [106]. Wang et al. meta-analyzed 20 studies that adopted DSC and found that the pooled sensitivity and specificity were 0.83 and 0.83 respectively, while the AUC was 0.89 [104]. A more recent study by Tsakiris evaluating the differentiation of true tumor progression versus pseudoprogression using a random effect model in five DSC studies found there to be a pooled sensitivity and specificity of 0.81 and 0.82 [107]. Some studies also found that certain histographic patterns are the best independent predictors of tumor recurrence [87]. CBV is also lower in bevacizumab-induced abnormalities than in recurrent tumors [76]. A few publications also investigated other DSC parameters. For example, PH and PSR were found to be respectively increased and decreased in tumor recurrence [93].

DCE was more scarcely assessed but provided analogous results [82,88,95,96]. Ktrans has been found to be consistently higher in tumor progression/recurrence as opposed to radiation necrosis [82,88,95]. The same pattern was observed for iAUC [82,88]. A more detailed AUC analysis methodology was also applied by Suh et al. who could, with high sensitivity and specificity, differentiate between the two entities [99]. Vp and Ve were reported to be higher in progressive lesions versus radiation necrosis and pseudoprogression [95,96], while other reports found there to be no significant difference [88]. Pooled sensitivity and specificity were confirmed through a series of meta-analyses. Tsakiris et al. also evaluated performance in a random effect meta-analysis of DCE in the differential diagnosis between tumor progression and pseudoprogression. They found there to be a pooled sensitivity and specificity of 0.88 and 0.77, respectively [107]. In their meta-analysis including four DCE studies, Wang et al. found a pooled sensitivity and specificity of 0.73 and 0.80, and an AUC of 0.94 [104]. Okuchi et al. meta-analyzed nine studies for a total of 179 TR and 119 treatment-related changes. They found there to be a pooled sensitivity and specificity of 0.88 and 0.86, respectively, and an AUC of 0.89 [108]. ASL has been less investigated. Despite Myoshi et al. finding no correlation with tumor recurrence [109], another study by Nyberg et al. showed ASL-CBF to be more sensitive than standard imaging for identifying tumor progression in patients treated for high-grade glioma [110]. In the three studies assessing ASL included in Wang’s meta-analysis, the pooled sensitivity and specificity were 0.79 and 0.78, respectively, and the AUC was 0.89 [104]. PCT studies on a few patients have also shown that CBV and CBF are increased in tumor recurrence, while mean transit time (MTT) is decreased [111,112]. The PS was also significantly decreased regarding the treatment-induced changes [111].

### 2.8. Prognosis Prediction

In the treatment of diffuse cerebral glioma, a precise stratification of outcome can support the multidisciplinary team caring for the patient, strengthening the evidence-based therapeutic decisions. Currently, a prognostic evaluation is based on clinical factors, standard imaging, and histopathological-immunochemical tissue analysis [3]. Ultimately, hemodynamic imaging-based differential diagnosis, the prediction of grading, molecular features, and the differentiation of recurrent disease from the treatment effects, if proven with strong evidence, will all have a prognostic correlation. Before any inferences about prognosis can be drawn, the identification of an association requires sound methodology [113]. In this section, the associations of hemodynamic imaging-derived parameters to prognostic outcome prediction, such as a response to treatment, progression free survival (PFS) [24,33,50,114,115,116,117,118,119,120,121], and overall survival (OS) [20,23,24,122,123,124] are reviewed. As a general rule, pre-treatment increased tumor vascularity and BBB leakage (and related perfusion and permeability parameters) correlate with tumor malignancy and invasiveness. As a result, they are correlated with a worse prognosis while response to chemoradiotherapy has been associated with a decrease in tumor perfusion and BBB permeability. Increased DSC-CBV was found to be predictive of decreased progression free survival [20,116,117,118,119,125,126,127] and decreased OS [20,71,117,119,122,123,124,125,128,129,130,130,131,132,133,134] in diffuse cerebral gliomas. These parameters can be also used to follow-up on lower grade glioma lesions and to monitor treatment effects [122,135,136]. An increase in DSC-CBV during follow up of low-grade gliomas has, for example, been suggested to predict malignant transformation [137]. Moreover, an increase in CBV after chemoradiotherapy was also found to be a predictor for decreased OS [102]. In accordance with this, the clinical trials on recurrent glioblastoma prospectively evaluating perfusion MRI found that a decrease in CBV after treatment correlated to increased OS and increased CBV with respect to the baseline was instead associated with decreased OS [138]. In another trial, low CBV pre-treatment was predictive of an early response to bevacizumab and improved OS [139]. DSC-CBF has also shown a trend of significance for predicting time to recurrence but has been less commonly investigated. With respect of the DCE studies, increased Ktrans was shown to be associated with decreased PFS [140,141,142,143] and OS [125,143,144,145,146] in several studies. Of specific interest, the baseline elevated Ktrans in non-enhancing T2 lesions has been independently associated with negative PFS [141]. In accordance, a study by Kickingereder et al. in 2015 reported that recurrent GBM patients with lower baseline Ktrans and higher voxel wise reductions were characterized by increased PFS and OS [147]. Similarly to DSC derived measures, DCE-derived Ktrans is found to decrease significantly after chemoradiotherapy [148,149] and anti-angiogenic treatment [139,150], and to correlate with OS [148]. A high post-treatment Ktrans, accordingly, predicts decreased PFS [142]. Møller et al. used DCE to assess the CBF changes during chemoradiotherapy. They reported that it increased early on in the treatment only to decrease to a level lower than the baseline after the treatment ended. Neither of these changes nor the baseline values were determined to be correlated to PFS [151]. Lower Kep was found associated with a favorable response to bevacizumab in recurrent high-grade glioma, and increased Kep was on the contrary associated with shorter PFS and OS [114,152], even if these observations have been not confirmed in other reports [40]. Increased Ve was also reported to be associated with decreased PFS [120] and OS [40,114] with increasing values during treatment also negatively affecting the prognosis in DIPG [125]. As with CBV and Ktrans, a decrease in Ve has been identified as able to monitor radiochemotherapy [149] and antiangiogenic treatment effects on tumor vasculature [150]. Increased Vp was also associated with decreased OS [145] together with increased AUC, [40] with some authors reporting there to be a correlation of the latter with lower survival only in MGMT unmethylated tumors [153]. On the whole, as additionally confirmed by the meta-analysis conducted by Choi et al., the decrease in perfusion parameters derived from DSC and DCE has been shown to be useful in monitoring antiangiogenic treatment effects even with the caveat of not necessarily translating to a better prognosis [154]. ASL studies showed that CBF is a negative predictor of PFS [33,155] and OS [23]. Lower CBF also showed a trend for increased time to recurrence, despite not reaching statistical significance in a study by Qiao et al. [33]. A few PCT studies reported there to be an association between the perfusion parameters and prognosis. Increased PCT-derived CBV and decreased permeability area product were found to be associated with decreased OS [156,157,158].

## 3. Future Directions

### 3.1. New Approaches to Hemodynamic Imaging

**Figure 2:**
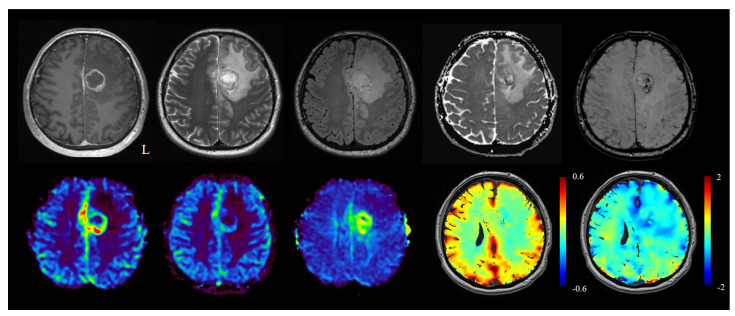
Standard MRI sequences, top row from left to right: T1 contrast-enhanced, T2, fluid attenuated inversion recovery (FLAIR), diffusion-weighted imaging (DWI), and susceptibility-weighted imaging (SWI). Perfusion MRI and advanced sequences, bottom row from left to right: CBV, CBF, MTT, BOLD-cerebrovascular reactivity (CVR), and hypoxia-enhanced BOLD MRI.

The perfusion parameters and permeability parameters allow for the description of the pathophysiological characteristics of abnormal vasculature in the tumor, yet the lesional histopathological heterogeneity suggests that a more refined hemodynamic and metabolic assessment of the glioma features could better describe tumor type, aggressiveness, and prognosis through an enhanced characterization of the microvascular hemodynamic habitat (Figure 2).

A few exemplary studies supporting this concept are now presented. Stadlbauer et al. applied a multiparametric MRI approach focusing on microvascular architecture including parameters such as microvascular density, vessel size index (VSI i.e., microvessel radius), neovascular activity (MTI, microvessel type indicator), and oxygen metabolism i.e., oxygen extraction fraction (OEF), cerebral metabolic rate of oxygen (CMRO2), and oxygen partial pressure (PO2) (by means of quantitative blood-oxygen level dependent (BOLD) MRI). In their series of publications, they show that this approach is able to predict glioblastoma recurrence through the identification of early pathophysiological alterations [159]. The same approach was used to identify different patterns of tumor microenvironment based on oxygen metabolism and neovascularization which can correlate with prognosis and phenotype switching [160,161]. As shown by the Garcia-Gomez group, the standardization of perfusion imaging and the correlation of imaging features can be integrated into standardized software-based ML-aided analysis for the determination of a refined “hemodynamic signature” (https://www.oncohabitats.upv.es/ Last accessed on 21 December 2021), i.e., of the microvascular habitat, as part of the identification of diffuse glioma regarding both the enhanced tumor and edema [134] with important prognostic implications [162,163] to integrate with other known molecular prognostic markers [62,164] and the potential to provide insight on treatment strategy refinements [165]. External validations of this pipeline, even if still based on modest sample sizes, have provided encouraging results [20,163] and support similar efforts in this direction.

### 3.2. Contrast-Enhanced Ultrasound (CEUS)

Intraoperative CE ultrasound (CEUS) has been also gaining attention for the intra-operative assessment of tumor vasculature [166]. This technique exploits special microbubble-based contrast agents to visualize the tumor vasculature. It is safe, repeatable, correlates to MRI findings of contrast-enhancement, [167] and it can be used to improve the extent of resection by identifying any residual tumor [166,168,169]. Moreover, through the means of perfusion assessment, the glioma grade can be predicted [170,171,172]. With respect of the techniques used to monitor CBF intra-operatively that are increasingly reported [173], these have been recently reviewed by Tahhan et al. [174].

### 3.3. Intravoxel Incoherent Motion (IVIM)-MRI

Based on a model proposed by Le Bihan et al. in the late 80s [175,176], IVIM-MRI was introduced as a diffusion-based method able to extract quantitative local microvascular perfusion information without the need for a contrast agent (see the review by Federau for further details) [177]. This technique yields three parameters, i.e., perfusion fraction (f) which is a measure proportional to CBV, pseudodiffusion coefficient (D *), and blood-flow related parameters fD *, and it has been the focus of brain tumor research as well. Similar to traditional hemodynamic imaging, IVIM-MRI has been also used for differential diagnosis (perfusion fraction is increased in high-grade compared with low-grade gliomas, and it allows for the differentiation of cerebral lymphomas—lower f versus high-grade glioma) [178,179,180], glioma grading, and IDH mutation prediction [181,182,183,184] to monitor the treatment effects [185,186,187], to identify tumor progression [109], and to predict survival [188,189,190]. 

### 3.4. Gas Modulation and BOLD Imaging: BOLD-CVR and Oxygen Modulation for Enhanced Lesion Characterization

The basic model underlying cerebrovascular reactivity has been addressed in Part A of the present review. As mentioned, in recent years, BOLD-cerebrovascular reactivity (CVR) has also been developed and intensively investigated in diffuse gliomas. Preliminary studies under breath-holding stimuli have revealed that the physiological cerebrovascular response is impaired in diffuse cerebral glioma patients possibly due to the altered vascular response in tumor tissue resulting in flow redistribution and the steal phenomenon [191,192]. CVR mapping has been also used to assess neurovascular uncoupling [193]. As previously introduced, the refinements in gas modulation control have provided better reproducibility for the evaluation of CVR [194,195]. Some of the recent studies exploiting this advantage have provided insights into glioma hemodynamic. CVR is in fact altered in areas of high-grade glioma recurrence [196] in the peritumoral tissue, with impairment confirmed by altered hemodynamic perfusion measures [197], and extending beyond the CE, FLAIR/T2, and hypermetabolism observed in positron emission tomography (PET) imaging [198]. Furthermore, BOLD-CVR has been able to elucidate remote changes in patients with gliomas such as a whole-brain decreased CVR suggesting global hemodynamic alterations [199] and the presence of crossed cerebellar diaschisis [200], and has shown the potential to distinguish radiation necrosis from glioblastoma [79]. Differential responses during separate oxygen and carbon dioxide modulation in the same tissue voxels may, in principle, hint at aberrant versus functional vasculature [201]. Interestingly, oxygen modulation has been also recently proposed as a means to study cerebral perfusion through rapid transient hemoglobin desaturation with the potential to substitute contrast-based perfusion [202,203,204] with advantages also relating to the avoidance of gadolinium contrast use [205], but application in the study of brain tumors is still lacking. Independently of these developments, the possibility of precise end-tidal oxygen modulation in isocapnic conditions has been preliminarily investigated as a means to provide a novel “imaging biomarker” to detail glioblastoma lesion microvascular features during BOLD imaging, exploiting hypoxia and hyperoxia as BOLD contrasts [206].

### 3.5. Machine-Learning and Radiomics

The new development of radiomics allows for the extraction of a high number of quantitative features to identify relations in the data that are not appreciable through traditional analytical methods [207,208]. Radiomics, and the possibility to combine it with machine learning algorithms, have shown considerable potential in terms of improving the diagnostic, prognostic, and predictive accuracy of conventional imaging analysis [207,208,209,210]. For example, every year, the Multimodal Brain Tumor Image Segmentation Benchmark (BRATS) challenge serves as a platform for developing better algorithms aimed at brain tumor segmentation. Other projects such as the REMBRANDT also make available genomics data of glioblastoma patients in conjunction with acquired imaging sequences annotated by expert neuroradiologists using the VASARI feature set [211,212]. This rapidly growing area of research has also adapted to using the data obtained from hemodynamic imaging modalities such as DSC [213], DCE [214], and ASL [215]. The use of perfusion imaging-derived radiomic features has shown promising feasibility in predicting MGMT promoter methylation [34] and IDH mutation status [216,217,218,219] and for improving the differential diagnosis of gliomas [220], the diagnostic performance of tumor grading [215,221] as well as pseudoprogression [222,223,224], but also prognostication [225]. Perfusional tumor heterogeneity can also be used to extract the radiomic features needed to train deep learning models for the prediction of glioblastoma recurrence patterns [226].

## 4. Conclusions

Advanced hemodynamic imaging research has provided yet another promising imaging development to complement traditional tumor grading by correlating the specific hemodynamic patterns posed by diffuse glioma to molecular and pathophysiological alterations. Despite promising associations being found, the evidence remains overall scarce and often conflicting with traditional histopathological and immunohistochemical analysis remaining the gold standard for diffuse cerebral glioma diagnosis. The monitoring of treatment effects and the differentiation of tumor progression/recurrence from treatment-induced changes can benefit significantly from hemodynamic imaging. The present literature points out the encouraging results for this purpose with the caveat that the lack of acquisition and processing standardization still limits its reliability for diffuse clinical integration. The identification of hemodynamic biomarkers correlated to prognosis can assist clinical management decisions and provide the basis for trial stratification to reveal missed patterns in connection to the treatment effects. As methodological optimization continues to be pursued, innovative hemodynamic imaging approaches are emerging with the potential to further advance the characterization of diffuse cerebral gliomas pre-operatively and during the follow-up by overcoming known technical limitations. The integration of radiomics and machine learning in the analysis pipeline can further extend the diagnostic and prognostic potential of hemodynamic imaging in cerebral diffuse glioma.
